# Variation in the population structure between a natural and a human-modified forest for a pioneer tropical tree species not restricted to large gaps

**DOI:** 10.1002/ece3.1528

**Published:** 2015-05-25

**Authors:** Milene Silvestrini, Flavio Antonio Maës dos Santos

**Affiliations:** 1 Programa de Pós-Graduação em Ecologia, Departamento de Biologia Vegetal, IB, Universidade Estadual de Campinas (UNICAMP)970, Monteiro Lobato, P.O. Box 6109, 13083-862, Campinas, SP, Brazil; 2 Departamento de Biologia Vegetal, IB, Universidade Estadual de Campinas (UNICAMP)13083-862, Campinas, Brazil

**Keywords:** Canopy gaps, disturbances, environmental heterogeneity, population structure, semi-deciduous tropical forests, succession

## Abstract

The distribution of tree species in tropical forests is generally related to the occurrence of disturbances and shifts in the local environmental conditions such as light, temperature, and biotic factors. Thus, the distribution of pioneer tree species is expected to vary according to the gap characteristics and with human disturbances. We asked whether there was variation in the distribution of a pioneer species under different environmental conditions generated by natural disturbances, and between two forests with contrasting levels of human disturbance. To answer this question, we studied the distribution patterns and population persistence of the pioneer tree species *Croton floribundus* in the size and age gap range of a primary Brazilian forest. Additionally, we compared the plant density of two size-classes between a primary and an early successional human-disturbed forest. *Croton floribundus* was found to be widespread and equally distributed along the gap-size gradient in the primary forest. Overall density did not vary with gap size or age (*F*-ratio = 0.062, *P *=* *0.941), and while juveniles were found to have a higher density in the early successional forest (*P *=* *0.021), tree density was found to be similar between forests (*P *=* *0.058). Our results indicate that the population structure of a pioneer tree species with long life span and a broad gap-size niche preference varied between natural and human-disturbed forests, but not with the level of natural disturbance. We believe this can be explained by the extreme environmental changes that occur after human disturbance. The ecological processes that affect the distribution of pioneer species in natural and human-modified forests may be similar, but our results suggest they act differently under the contrasting environmental conditions generated by natural and human disturbances.

## Introduction

Pioneer species are thought to present different population structures under different stages of forest succession or levels of human disturbances (Matthes 1992; Swanson et al. 2011; Tabarelli et al. 2012). In primary or late successional forests, species' population dynamics may resemble those defined for a metapopulation, that is, the populations persist at a regional scale as a result of a balance between the processes of local population extinction, patch migration, and colonization (Levins 1969; Hanski and Gilpin 1991; Freckleton and Watkinson 2002). In early successional forests, which have regenerated after human disturbances, the distribution patterns, survival, and reproduction of pioneer species are believed to change due to the shifts in the local environmental conditions, such as light, temperature, and biotic factors (Matthes 1992; Martínez-Garza and Howe 2003; Swanson et al. 2011; Tabarelli et al. 2012; Lohbeck et al. 2014). These expectations are based on the exploiter-mediated coexistence model in patches, which states that coexistence of tree species in tropical forests and the distribution of their populations rely on the differential availability of resources and space generated by gap disturbance regime and/or human disturbances (Connell 1978; Paine and Levin 1981; Begon et al. 2006; but see Hubbell et al. 1999). Thus, in a primary forest, the metapopulation structure of pioneer trees would be the result of the patchy recruitment generated by treefall gap disturbances, and, in an early successional human-disturbed forest, tree species would also respond to variation in resource availability by changing their demographic characteristics according to the new environmental conditions of the area.

Recent studies have found a much higher density of pioneer species under human-modified environments, such as the edges of tropical fragmented forests, when compared to the forest interior (Laurance et al. 2006; Santos et al. 2008, 2012). The forest edges would function as an early successional forest due to the similarity of environmental conditions in the areas, mainly elevated light availability (Tabarelli et al. 2008; Santos et al. 2012). Despite the consensus in the response among pioneer species regarding the juvenile abundance, long-lived pioneer species were found to exhibit populations with negative adult recruitment along the forest edges (Santos et al. 2012). According to Santos et al. (2012), the reason was probably the adult sensitivity to edge effects, such as wind and physiological stress. However, the existence of an underlying pattern for the distribution of long-lived and short-lived pioneer species under these conditions remains unclear. Furthermore, there are still few empirical data available from early successional forests.

In well-conserved or late successional forests, pioneer density can vary greatly among gaps, depending on the species (Brokaw 1987). It seems that some pioneer species have a narrower niche preference than others, such as *Trema micrantha* (L.) Blume and *Trema orientalis* (L.) Blume (Ulmaceae). These species occupy preferably, have high plant density, and reproduce only in large gaps (above 400 m^2^) or in high light environments (Brokaw 1987; Goodale et al. 2012). The higher plant density in large gaps and in edge-affected habitats and/or early successional forests is thought to be a result of the fine specialization to the high availability of resources, mainly light, in these areas by these short-lived, rapid-colonizing pioneer species (Brokaw 1987; Goodale et al. 2012). These high resource levels should sustain the higher and faster growth of these species (Bazzaz and Pickett 1980; Brokaw 1987). In contrast, some pioneer species seem to be able to survive and reproduce across wider ranges of gap size and light conditions (Brokaw 1987; Goodale et al. 2012), which is the case of *Miconia argentea* (Sw.) DC. (Melastomataceae), a relatively “shade-tolerant” pioneer species that is found in a broad range of gap sizes (from 100 m^2^ to 705 m^2^), and therefore has a large-gap-size niche preference (Brokaw 1987).

High density of large-gap-specialized species in large gaps or open habitats may also be the result of an increased availability of other resources, such as soil nutrients and water (Engelbrecht et al. 2007; John et al. 2007). Additionally, biotic interactions may play a role, as these large-gap-specialized species appear unable to deal with the intense herbivory, and the need of a high production of new leaves under low irradiances (Salgado-Luarte and Gianoli 2010; Goodale et al. 2014). On the other hand, resource competition with lianas, grass, and weeds could be an important ecological filter for pioneer tree species, decreasing plant density, especially in smaller gaps and after severe human disturbances (Uhl et al. 1991; Holl et al. 2000; Schnitzer et al. 2005; Toledo-Aceves and Swaine 2008).

**Figure 1:**
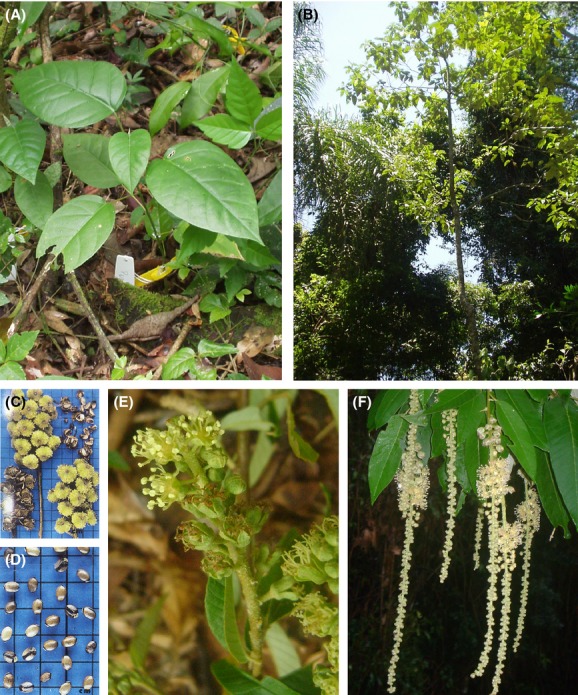
*Croton floribundus* Spreng.: A juvenile (A) and a young tree (B) growing in canopy gaps of the primary forest. Fruits (C) and seeds (D), source: Lorenzi (1992). Feminine (E) and masculine (F) flowers of the species.

*Croton floribundus* Spreng. (Euphorbiaceae) (Fig. 1) is a shade-intolerant, fast-growing pioneer tree species (Lorenzi 1992; Gandolfi et al. 1995; Rodrigues 1995) that presents high photosynthetic rates (M. Silvestrini and I. F. M. Válio, unpubl. data; Ribeiro et al. 2005) and germinates only under alternating temperatures (Válio and Scarpa 2001). It is abundant in gaps of primary remnants as well as in secondary areas of the semi-deciduous tropical forest (Lorenzi 1992; Gandolfi et al. 1995; Rodrigues 2005). These features seem to be directly related to a narrow niche preference: large gaps or high light environments (Pearson et al. 2002). Thus, one can expect higher densities of both reproductive and juvenile individuals of this species in early successional habitats rather than in natural or primary forests. Likewise, both size-classes should be found mostly in the large gaps of the primary forest, because only at these sites would there be the level of resources required by the rapid growth of *C. floribundus*. However, the species presents some unusual pioneer tree characteristics, such as autochorous seed dispersal syndrome (Lorenzi 1992), no seed banks (Lorenzi 1992; Grombone-Guaratini 1999; Carvalho 2001; Grombone-Guaratini et al. 2004) and no asexual reproduction (Passos 1995; Danciguer 1996), which demonstrates that it may have a different ecological behavior from the traditionally studied pioneer species restricted to large gaps. In addition, this species was found to be higher and have a larger diameter in a primary forest (Rodrigues 2005; T. E. Barreto, unpubl. data) than in secondary forests (Lorenzi 1992; Danciguer 1996). This suggests that *C. floribundus* may be a long-lived pioneer species and not short-lived, as it has been generally considered (Lorenzi 1992; Danciguer 1996).

Here, we evaluated the distribution of *C. floribundus* individuals in the gaps of a primary forest and compared the plant density of two size-classes between a primary and an early successional forest to understand the pioneer plant distribution under the variable environmental conditions generated by natural or human disturbances. Additionally, we examined the population persistence of the species and the relation between size-class and reproductive activity in the primary forest, where larger sized plants were observed compared with secondary forests. The following questions were addressed: (1) Does *C. floribundus* have a large, intermediate, or narrow gap-size niche preference in the primary forest? (2) Does the pioneer species present a similar plant density between natural and human-modified environments of a primary and an early successional forest? (3) Is there difference in the space occupation of juveniles and trees of the pioneer species between forest types? (4) Is *C. floribundus* a long-lived pioneer species?

## Materials and Methods

### Study site

**Figure 2:**
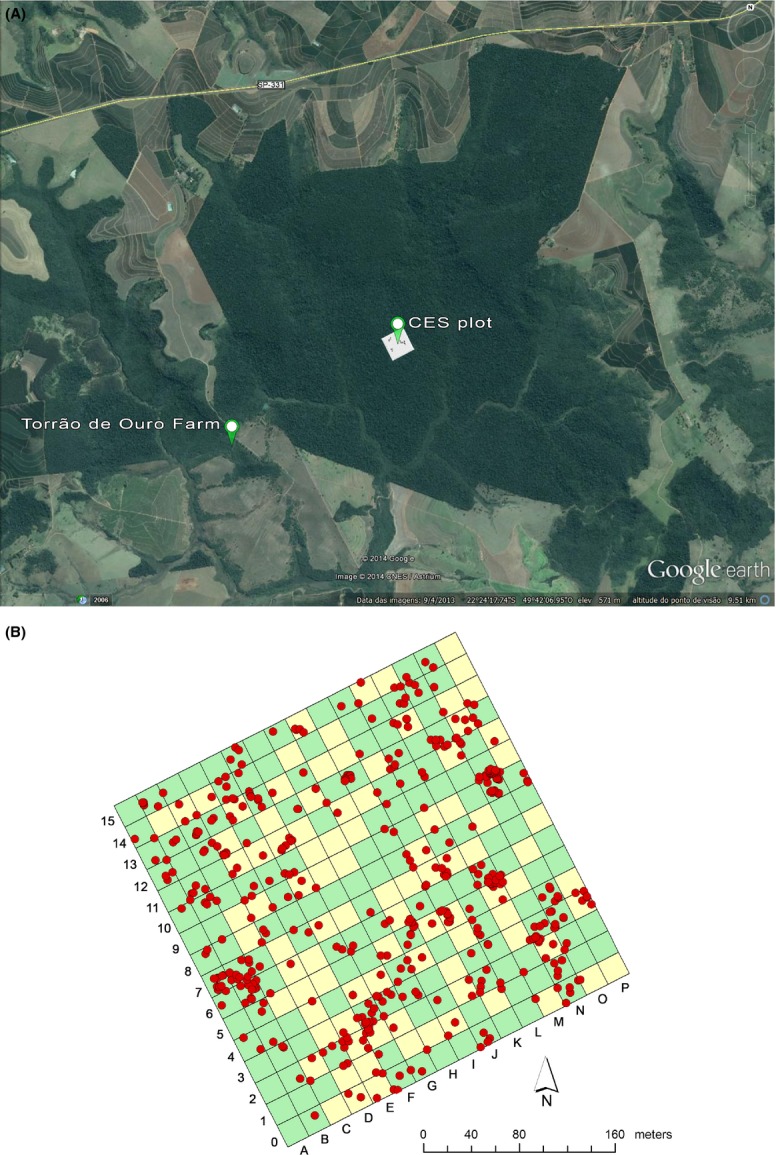
Location of the study sites: the permanent plot in the primary forest at Caetetus Ecological Station (CES plot) and the early successional forest at Torrão de Ouro Farm (A) (Satellite imagery © 2012 MapLink/Tele Atlas, GeoEye Image, via Google Earth). Map of the permanent subplots (20 × 20 m) at CES showing the subplots (in yellow) where the 100 canopy gaps recorded by Lima et al. (2008) and A. M. Z Martini and R. A. F. Lima (unpubl. data) were located and the trees (DBH ≥ 4.78 cm) of *Croton floribundus* Spreng. (red circles) censused by Rodrigues (2005) (B).

The study was conducted in a 10.24-ha permanent plot in a primary forest at Caetetus Ecological Station (CES) (Rodrigues 2005), and in an early successional forest adjacent to the state reserve at Torrão de Ouro Farm (Fig.2A). CES is located in Gália and Alvinlândia, state of São Paulo, Southeast Brazil (22°20′–22°30′S; 49°40′–49°45′W). Altitude ranges from 520 to 680 m (Tabanez et al. 2005). The climate at CES corresponds to Köppen's “Cwa” mesothermic type, that is, humid subtropical with a dry winter (Rodrigues 2005; Tabanez et al. 2005). Annual rainfall averages 1431 mm year^−1^, and average annual temperature is 21.5°C.

Caetetus Ecological Station consists of 2178.84 ha of semi-deciduous tropical forest, or the premontane moist forest, according to Holdridge (1967). This forest originally covered nearly the entire plateau in the state of São Paulo. It is currently the most threatened forest in São Paulo State due to past fragmentation and deforestation that has occurred since the beginning of European colonization in Brazil (16th century). Most of the area of CES is covered by well-conserved vegetation. The forest at CES is very dynamic, with larger gaps and gap density, and total gap area percent that is higher than other tropical forests (Lima et al. 2008; Martini et al. 2008). According to historical reports dating from the early 20th century, most of the area including the center of the reserve, where the 10.24 ha permanent plot was delimited, has not experienced anthropogenic disturbances. On the other hand, the edges of the reserve are highly disturbed (Tabanez et al. 2005). At the Torrão de Ouro Farm site, the original forest was cleared in 1926 and converted to a pasture. In 1984, the site was abandoned and farmers stopped slashing, but cattle grazing was still allowed. In 1986, the vegetation was burned, and in 2008, the pasture was completely closed to cattle grazing, allowing forest recovery. At the time, this study was conducted (2010), the site was already covered by an early successional forest. We define an early successional forest as an area that had originally been forested, but was then completely deforested to be used for human activities for a period of time before being abandoned and allowed to regenerate, with typical dominance by few fast-growing pioneer species. This type of forest differs from a primary forest mainly due to: (1) lower number of species (dominance of pioneer tree species); (2) lower canopy height; and (3) openness and discontinuity of the canopy, which contribute to the formation of a more illuminated understory.

Soils in the early successional forest at Torrão de Ouro Farm are of the same type as at the CES plot, that is, the red-yellow Acrisols (Ultisols) and Gleysols (Entisols) (Rodrigues 2005; M. Cooper, pers. comm.). Soil fertility is high and similar between forests (Silvestrini 2014).

### Study species

*Croton floribundus* Spreng. (Euphorbiaceae) (Fig. 1) is recognized as a pioneer species by Rodrigues (1995) and Gandolfi et al. (1995). It is a polyploid (Silvestrini et al. 2013), shade-intolerant, fast-growing tree species commonly found in the gaps of primary forest remnants, as well as in secondary areas of the semi-deciduous tropical forest (Lorenzi 1992; Gandolfi et al. 1995; Rodrigues 2005). The species range includes other forests in Brazil and in Eastern Paraguay (Lorenzi 1992; Gandolfi et al. 1995; Rodrigues 2005; Gomes 2006). According to Lorenzi (1992) and Danciguer (1996), tree height ranges from 4 to 13 m, but in the primary forest at CES, tree height ranges from 3 to 30 m (Rodrigues 2005; T. E. Barreto, unpubl. data).

The seeds (5 × 4.5 mm, 39.95 mg dry mass) (Fig. 1D) are much larger than other pioneer species and require alternating temperatures for germination in both light and darkness (Válio and Scarpa 2001; Gomes 2006). Seed dispersal occurs by autochory or ballochory, that is, explosive seed dispersal (Lorenzi 1992). The number of *C. floribundus* viable seeds found in soil banks was very low (1–5 seeds) compared, for instance, with other pioneer *Croton* species sympatric to *C. floribundus*, such as *C. priscus* Croizat and *C. urucurana* Baill. (13–77 seeds) (Grombone-Guaratini 1999; Carvalho 2001; Grombone-Guaratini et al. 2004). In addition, data from Lorenzi (1992) and M. T. Grombone-Guaratini (unpublished) show that seeds of *C. floribundus* present a short period of viability – 3–4 months, at the most. This plus the fact that *C. floribundus* seeds are heavily predated on (Carvalho 2001) leads us to assume that the ability of this species to persist long term in the soil seed bank is very limited.

For this study, the following size-classes based on Danciguer's (1996) definition of life stages for *C. floribundus* were used: (1) seedlings (presence of cotyledonary leaves); (2) juveniles 1, hereafter referred to as juveniles, which consisted of individuals of 0.05–0.40 cm diameter at soil height (DSH) (Fig. 1A); and (3) juveniles 2, which consisted of individuals of 0.40 cm DSH to 4.78 cm diameter at breast height (DBH) (Fig. 1B); (4) trees, which comprised individuals ≥4.78 DBH. The definition of tree size-class was based on the criterion of inclusion of the individuals in the available plant inventory from the permanent plot (DBH ≥ 4.78 cm) (Rodrigues 2005). Plant materials from two reproductive trees from CES and one from Torrão de Ouro Farm were deposited at UEC Herbarium, University of Campinas, Campinas, Brazil (accession numbers 172052, 172054, and 172055, respectively).

### Data analysis

#### Distribution of individuals in the gap range

Analyses of the distribution of *C. floribundus* individuals in the gaps of the primary forest were based on the previous gap characterization in the permanent plot, which was carried out by Lima et al. (2008), Martini et al. (2008), and A. M. Z Martini and R. A. F. Lima (unpubl. data) (Fig.2B). Gap area data were based on the method of Brokaw (1982): “the vertical projection of the hole in the forest extending through all levels down to an average height of two meters above ground”. Gap age classes were defined by Martini et al. (2008) as follows: Age 3 (old) = tree disturbance occurred before 2002 (>8 years); Age 2 (intermediate) = tree disturbance occurred after 2002 (≤8 years – intermediate); Age 1 (fresh) = tree disturbance occurred after 2002, but it is more recent than age 2 due to the presence of “bark and thin twigs on the terminal branches of the tree or the part of the tree (stem or branch)” (<8 years – fresh) (for further details, see Martini et al. 2008). The gap age in years was calculated from the year 2010, which was the year this study was conducted. We sampled sixteen gaps with different areas, ages, and modes of disturbance, in which density and frequency of *C. floribundus* individuals in all size-classes were evaluated. The range in gap size was from 23 m^2^ to 645 m^2^, and the total gap area sampled was 3578 m^2^.

As the distribution of size-class frequencies of canopy gaps followed the same pattern found by Lima et al. (2008) (data not shown), we considered our sampling a representation of the plot canopy gaps. Seven complex gaps, that is, gaps formed by distinct episodes of tree mortality, were evaluated. In these cases, disturbance age was based on the most recent episode.

A multiple regression was applied to test whether gap area (*X*_1_) and gap age class (*X*_2_) explained plant density (*Y*) in gaps (Zar 2010). The initial analysis included individuals of all size-classes. Subsequent analyses were then applied to each size-class (juveniles and trees) separately.

#### Population persistence

Population persistence of *C. floribundus* was inferred based on the size distribution of trees that had been previously recorded in the permanent plot in 2005 (*n* = 448, Rodrigues 2005) and 2010 (*n* = 509, T. E. Barreto, unpubl. data). Size distribution of individuals was visualized through box plots.

#### Reproductive activity and tree size

To evaluate the relationship between size and reproductive activity of *C. floribundus* trees in the primary forest, we observed the presence of flowers in 131 trees in the permanent plot at the beginning of December 2010, during the blooming period of the species (Lorenzi 1992; Passos 1995).

Simple logistic regression (Zar 2010) was used to calculate the probability of encountering a flowering tree at a given DBH in the population.

#### Plant density

Both juvenile and tree density (individuals m^−2^) of *C. floribundus* were assessed in the early successional forest at Torrão de Ouro Farm to compare them with the densities recorded in the primary forest at CES. All juveniles and trees found within eight transects of 2 × 50 m that were spaced 40 m apart, and distributed throughout the forest, were censused (a total area of 800 m^2^). In the CES permanent plot, juvenile density in the gaps was calculated based on the *C. floribundus* distribution survey in the sixteen gaps from the gap range analysis (a total Brokaw gap area of 3578 m^2^). For trees, density was obtained by dividing the number of individuals surveyed in each permanent subplot (T. E. Barreto, unpubl. data) by the subplot area (400 m^2^) (*n* = 256) (a total plot area of 102,400 m^2^). As the sampled populations were asymmetrical, and sample sizes differed, mainly for trees, we did not perform tests for assumptions of the parametric statistical tests, as recommended by Zar (2010). Nevertheless, we did report the variability for each of the samples. Both parametrical and nonparametrical tests were performed (two-sample *t*-test with separate variances and the Mann–Whitney test, respectively).

The simple logistic regression was performed using software R version 2.15 (R Development Core Team, 2010). All other statistical analyses were performed using Systat 11 software (Systat Software Inc., Richmond, CA).

## Results

### Distribution of individuals in the gap range

**Figure 3:**
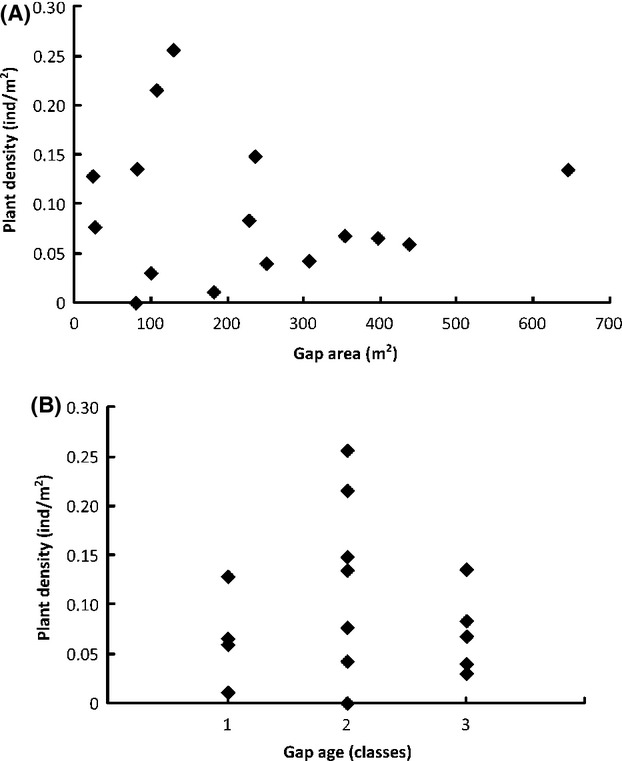
Scatter diagram of plant density (all size-classes) of *Croton floribundus* Spreng. (ind m^−2^) as a function of Brokaw gap size (m^2^) (A) and gap age class (B) in sixteen canopy gaps in the permanent plot at Caetetus Ecological Station (CES). Age 3 = before 2002, Age 2 = after 2002, Age 1 = after 2002, but more recent than age 2 (see more details in the text).

A total of 317 individuals in all size-classes of *C. floribundus* were found in the sampled gaps of the primary forest. Frequency of *C. floribundus* in the gaps was 94%. The species was found in all gap sizes (Table1, Fig.3A), except in gap H9 (Table1, Fig.2B). A possible explanation for this could be that while this gap has disturbance modes, gap area, and gap age (Table1) similar to the other gaps analyzed, it was not surrounded by any *C. floribundus* trees within a radius of 34 m (Fig.2B).

**Table 1:** Plant density of *Croton floribundus* Spreng. (ind m^−2^) in sixteen canopy gaps in the permanent plot at Caetetus Ecological Station (CES). Age 3 = before 2002, Age 2 = after 2002, Age 1 = after 2002, but more recent than age 2 (see more details in the text)

Gap	Age	Brokaw area (m^2^)	Plant density (ind m^−2^)			
			Seedlings	Juveniles	Juveniles 2	Trees
D12	3	353	0	0.003	0.034	0.031
D5^1^	2	107	0	0.206	0.009	0
D7^1^	1	396	0.005	0.015	0.045	0
E11	3	81	0	0.062	0.074	0
F11	3	251	0	0.004	0.012	0.024
F7^1^	2	236	0.064	0.042	0.034	0.008
F9^1^	2	306	0	0.033	0.010	0
G10	1	23	0	0.086	0.043	0
G11	3	228	0	0.066	0.018	0
G7^1^	1	437	0	0.007	0.048	0.005
H9	2	80	0	0	0	0
H10	2	26	0	0.038	0.038	0
N8	2	129	0	0.039	0.218	0
P10	3	99	0	0	0	0.030
P11^1^	1	182	0.011	0	0	0
P3/P4^1^	2	645	0.059	0.050	0.022	0.005

1 Complex gaps (gaps with recurrent tree disturbances).

**Figure 4:**
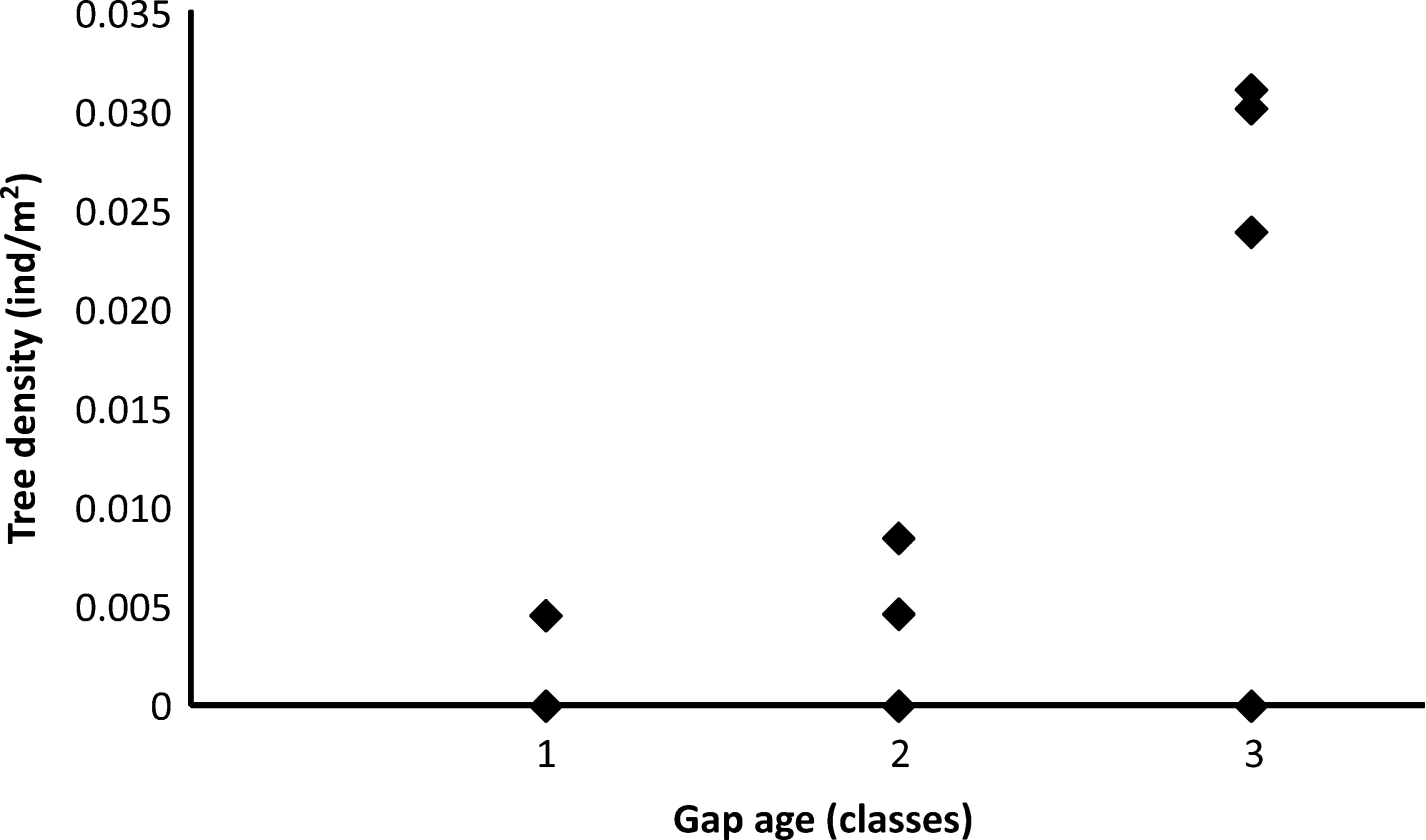
Scatter diagram of tree density (DBH ≥ 4.78 cm) of *Croton floribundus* Spreng. (ind m^−2^) as a function of gap age class in sixteen canopy gaps in the permanent plot at Caetetus Ecological Station (CES). Age 3 = before 2002, Age 2 = after 2002, Age 1 = after 2002, but more recent than age 2 (see more details in the text).

Density of *C. floribundus* (all size-classes) in the gaps was independent of gap size and gap age (*F*-ratio = 0.062, *P *=* *0.941) (Fig.3). However, while juvenile density did not vary with either gap area or gap age class (*F*-ratio = 0.435, *P *=* *0.657), we found a higher density of trees (DBH ≥ 4.78 cm) in older gaps (age 3) (*P *=* *0.02, two-tail, gap age coefficient) (Fig.4). Juveniles of *C. floribundus* were recorded even in old gaps, that is, gaps more than 8-year-old (Table1). However, this occurred when trees were not present in the gaps. In gaps that contained trees, there were no seedlings and a very low number of juveniles (≤1 individual), except for gaps where there were recurrent tree disturbances (Table1). The highest DBH recorded in a gap was of 20.85 cm.

### Population persistence

**Figure 5:**
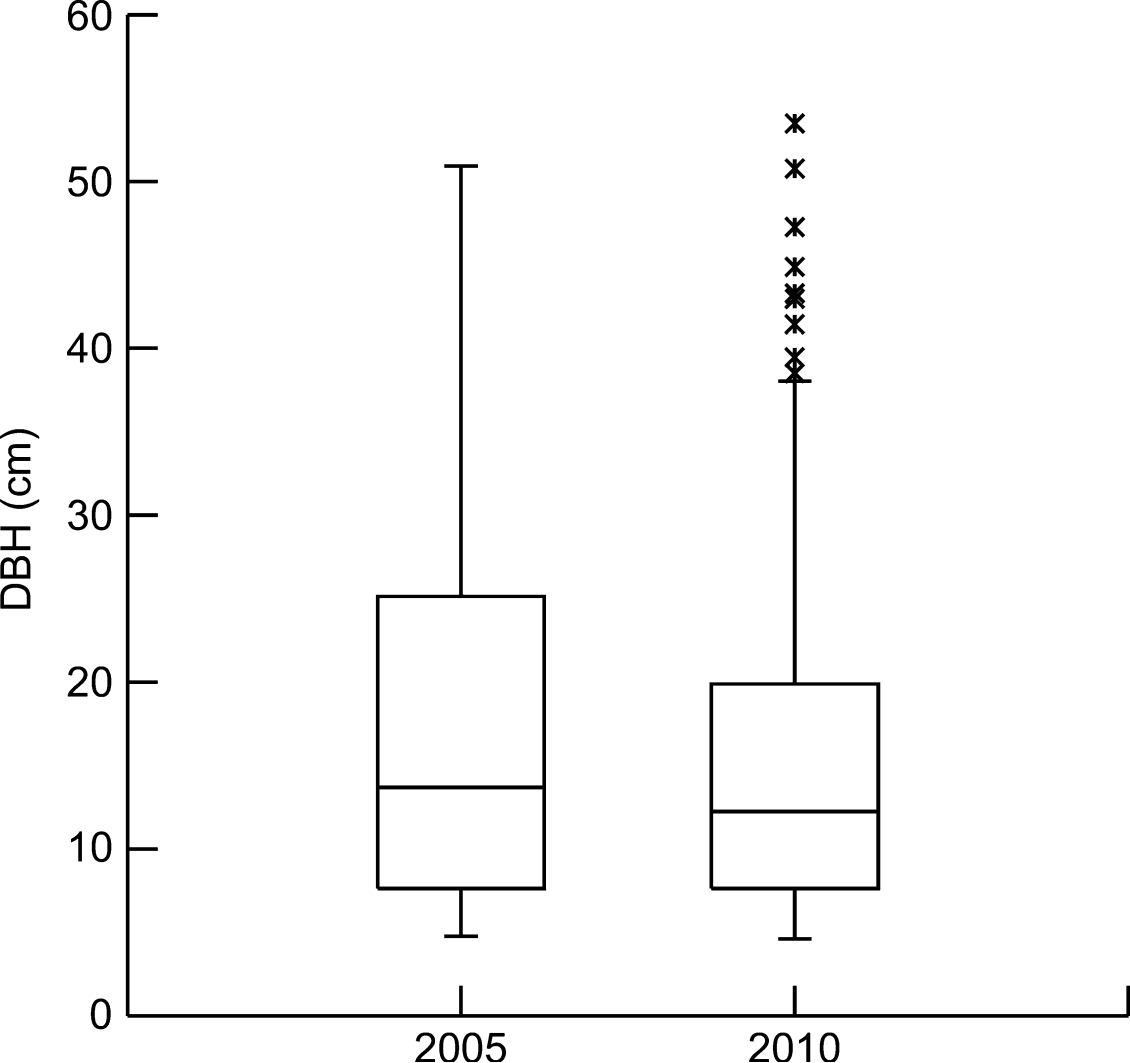
Box plot of tree DBH of *Croton floribundus* Spreng. in the permanent plot at Caetetus Ecological Station (CES) censused in 2005 (*n* = 448, Rodrigues 2005) and 2010 (*n* = 509, T. E. Barreto, unpubl. data). The length of each box shows the range within which the central 50% of the values fall, with the box edges at the first and third quartiles. * = outliers.

The DBH ranged from 7.9 to 20.0 cm for the majority of trees found in the permanent plot of the primary forest at CES (Fig.5). However, at least 25% of the individuals were larger than 20.0 cm DBH. In addition to the high abundance in the populations, these larger trees showed a tendency of a lower growth rate than that of the small and average size individuals (Appendix 1). This indicates that populations of *C. floribundus* can persist for relatively long periods of time after gap disturbances in the primary forest and that the species has a relatively long life span.

### Reproductive activity and tree size

**Figure 6:**
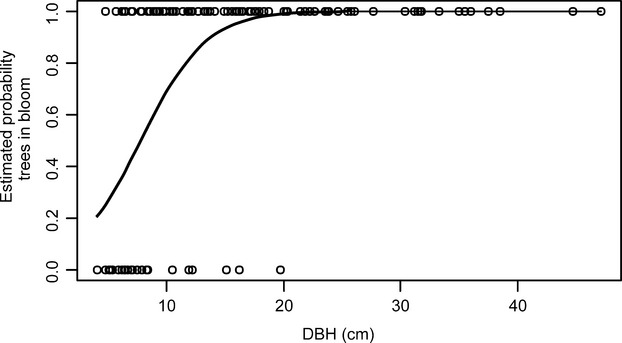
Logistic regression of trees in bloom on DBH (cm) for *Croton floribundus* Spreng. in the permanent plot at Caetetus Ecological Station (CES).

Flowering individuals were found in all evaluated sizes (from trees with 4.78 cm to 47.11 cm DBH) (Fig.6). The estimated linear logistic model was the following:

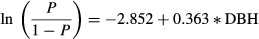
where *P* is the probability of encountering a plant in bloom at a given size. Both logit coefficients were significant with *P *≪ 0.0001. The chances of reproduction were 90% for trees with DBH ≥ 13.9 cm and 99% for trees with DBH ≥ 20.5 cm (Fig.6).

### Plant density

Density of trees did not differ (*P *=* *0.058) between the early successional forest at Torrão de Ouro Farm (0.023 ± 0.008 ind m^−2^, *n* = 8) and the primary forest at CES (0.0050 ± 0.0005 ind m^−2^, *n* = 256). The Mann–Whitney test also showed statistical similarity of tree density between both forest types (*P *=* *0.058). Juveniles presented higher densities (*P *=* *0.021) in the early successional forest at Torrão de Ouro Farm (0.21 ± 0.06 ind m^−2^, *n* = 8) than in the gaps of the CES primary forest (0.04 ± 0.01 ind m^−2^, *n* = 16). A similar statistical result was found by the Mann–Whitney test (*P *=* *0.003).

## Discussion

We found *C. floribundus* to be widespread and equally distributed along the gap-size gradient in the primary forest, which is in contrast to what would be expected based on the ecophysiological characteristics of the species. Furthermore, population persistence inferred by the size distribution of trees indicated that this species might be long lived under natural conditions and might reach reproductive stage at smaller size-classes (Poorter et al. 2006; Bentos et al. 2008). The study species showed a regeneration behavior similar to the pioneer species *Miconia argentea* (Sw.) DC. (Melastomataceae), and opposite to *T. micrantha* and *T. orientalis* (Brokaw 1987; Goodale et al. 2012). These two groups of species are considered as the “endpoints on a scale of regeneration behavior” (Brokaw 1987). *Croton floribundus* and *M. argentea* seem to have other similar characteristics, such as plant recruitment and persistence for a long time after the creation of a gap; that is, colonization process can persist for years after gap formation. We found juveniles being recruited in old gaps, and it was common to find seedlings, juveniles 1, and juveniles 2 (early and late recruits) coexisting in gaps, but we did not find trees coexisting with seedlings and juveniles. It is worth noting that there was a large variation in the size of trees in old gaps, which we believe is likely the result of colonization occurring over an extended period of time. For instance, we found plants ranging from 4.78 cm to 15.60 cm DBH in a gap and 9.07–20.85 cm DBH in another.

During the gap analysis, we verified that *C. floribundus*, mainly seedlings and juveniles, was also present under the canopy trees surrounding the openings. This, along with the results discussed above, may demonstrate that *C. floribundus* has a higher shade tolerance than pioneer species commonly restricted to large gaps. This finding also provides evidence of its broad gap-size niche preference. Thus, this species seems to occupy a more heterogeneous environment. Such characteristics confirm that, as other regeneration classes of trees, pioneer species can also show a continuum of responses to irradiance (Brokaw 1987).

Despite the high forest turnover rate for the semi-deciduous tropical forest at CES (Lima et al. 2008) – 98 years considering the Brokaw (1982) area method and 38 years considering the Runkle (1982) area method – and the high frequency of recurrent disturbances (Lima et al. 2008), population persistence of *C. floribundus* is high due to the survival of a few old remnant individuals in each gap. The survival of large and slow-growing *C. floribundus* individuals (Fig.5, Appendix 1) and the apparent high shade tolerance show some similarity to the pioneer species, *Alseis blackiana* Hemsl. (Rubiaceae). This species requires gaps for establishment, but its saplings and trees present a high shade tolerance and very low mortality rates in the understory (Dalling et al. 2001). The unusual life history of *A. blackiana* was characterized as a rare regeneration strategy in tropical forests (Dalling et al. 2001); however, the results found in the present study, as well as results from other pioneer tree species (Poorter et al. 2005), indicate that this kind of response, that is, broader patterns of gap-phase regeneration behavior through ontogeny, might be more common than originally believed.

The long life span of *C. floribundus* seems to be associated with an extended reproductive phase, as reproduction may begin at early life stages or small size-classes. On the other hand, earlier reproduction may be a selective advantage for a light-demanding pioneer species that can grow in small or medium gaps, where rapid canopy closure can decrease chances of plant survival in later life stages. Furthermore, an extended period of seed production can result in a higher number of seeds per individual. This could overcome the short-distance seed dispersal by helping the species reach gaps in time instead of space. Such a strategy would be consistent with a large-gap-size niche preference and a high forest turnover rate because any new opening gap would be a favorable regeneration site for the pioneer species.

The density of *C. floribundus* individuals in all size-classes in the gaps of the permanent plot was found to be unpredictable as a function of gap size and age, but an association was found between gap age and the abundance of trees (DBH ≥ 4.78 cm). This means that we cannot expect high plant densities in the early successional forest, as we would expect in a large gap or a high light environment (Brokaw 1987; Goodale et al. 2012; Santos et al. 2012). Indeed, even though the early successional forest has favorable light and temperature microclimate conditions for pioneer recruitment, especially in the beginning stages of colonization, tree density was found to be similar between the forest types. An explanation for this could be the low number of colonizers in the area (Martínez-Garza and Howe 2003). The large vacant site prior to the regeneration of the early successional forest seems not to be easily reached by a short-distance dispersal tree, such as *C. floribundus* (Stamp and Lucas 1983; Lorenzi 1992; Passos and Ferreira 1996). Furthermore, there might be plant recruitment and establishment barriers as a result of past land use and modified ecological conditions, such as cattle grazing (see land use history) and competition with grasses and weeds (Uhl et al. 1991; Holl et al. 2000). The long-term persistence of *C. floribundus* in the primary forest, and the age of the early successional forest, can account for the tree abundances in both forests, explaining the results as well. Our results are consistent with previous studies, which have shown that for some pioneer species, specifically long-lived ones, only saplings and juveniles had higher plant density in early successional or secondary environments (Danciguer 1996; Santos et al. 2012). Most importantly, the results showed that even for a pioneer species not restricted to large gaps, that is, whose distribution is not affected by the different environments in the gap-size range, the population structure changes after human disturbances.

Interestingly, one gap in the primary forest had a similar density of juveniles as in the early successional forest (0.206 ind m^−2^, gap D5, Table1). This gap had a large reproductive *C. floribundus* tree near the border of the Brokaw area providing seeds for colonization of the gap (Fig.2B). Also, other gaps that were adjacent to old gaps (A. M. Z Martini and R. A. F. Lima, unpubl. data) with reproductive *C. floribundus* trees, presented relatively higher juvenile density (for instance, gap G11, Table1 and Fig.2B). The opposite response was found in gap H9, which was not surrounded by any tree of *C. floribundus*. These results indicate that the main determining factors of first-colonizer abundance in both forest types may not be only gap area, or the suitable sites available for plant establishment, but also, the source of seeds and barriers of seed dispersal, such as the short-distance seed dispersal mechanism (Dalling et al. 2002). As mentioned above, this may also be an explanation for the absence of a relationship between gap area and plant density in the primary forest. Besides the limited seed dispersal, other barrier to seed arrival in the open gaps might be the presence of large trees and dense tangle of lianas in the primary forest (Rodrigues 2005; Lima et al. 2008).

Plants of different life stages, namely juvenile 1, juvenile 2 and reproductive *C. floribundus*, occurred spatially segregated in a 25-year-old secondary forest according to Danciguer (1996). In the primary forest analyzed in the present study, trees were apparently segregated from juveniles, but were not segregated from juveniles 2, while juveniles 2 were not spatially segregated from juveniles. In the early successional forest, juveniles occurred in the same transects as trees. The spatial structure of the species seems to be in accordance with the idea that there is a slow growth and an accumulation of juveniles in secondary forests, particularly in the early or mid-successional stages, due to the unfavorable growth conditions for this size-class, such as the shading of tree crowns (Danciguer 1996; Goodale et al. 2012). Despite its relatively high shade tolerance, and therefore the capability to survive under more shaded environments, *C. floribundus* still requires high light conditions for recruitment to higher size-classes. Conversely, in the primary forest, where gaps containing juveniles usually do not have trees of *C. floribundus,* or other pioneer species, there are light and temperature conditions for rapid growth, and thus, the transition from juvenile to juvenile 2 is facilitated. The accumulation of individuals in time in the early successional forest indicates that juveniles may have come from several different events of seed dispersal, as was found in the secondary forest studied by Danciguer (1996). In addition, there might have been an increase in the number of migrants by seeds after the initial colonization due to the removal of cattle and reduction in grass and weed competition, which would result in higher juvenile density. Thus, the specific biotic and abiotic conditions of the early successional forest may favor the survival of juveniles, but hinder their growth and transition to the next size-class.

Likewise, competition and herbivory may affect juvenile density in a different way in the primary forest. In general, juveniles from the early successional forest showed phenotypic characteristics very different from the primary forest, such as less freshness and more damaged leaves, presence of re-sprouts, and lower height (Appendix 2), which indicates more injuries and signs of recovery through re-sprouting. The re-sprouting ability in *C. floribundus* trees as well as branching in life-stage juvenile 1 (Danciguer 1996; Martini et al. 2008) might play an important role to increase the potential competitive ability and survival of the species. Thus, it is likely that individuals in new, highly human-disturbed environments use this ability to maintain growth, even at very slow rates, and survive herbivory under shadier conditions in the most critical life stages. Goodale et al. (2014) found high survival rates with low growth in more shade-tolerant pioneer species growing in shady environments. Furthermore, one of these species, *Wendlandia bicuspidata* Wight and Arn (Rubiaceae), responded positively to herbivory by re-sprouting (Goodale et al. 2014), which is similar to what has been found for *C. floribundus*. In the primary forest, on the other hand, the site-specific seedling predation/herbivory (Uhl et al. 1991) might be combined with resource competition with lianas in the gaps (Schnitzer et al. 2005; Toledo-Aceves and Swaine 2008), which could create a stronger negative effect on survival and growth, decreasing juvenile density. This statement is based on the high density of lianas found in the gaps of the permanent plot of CES (Lima et al. 2008), and our observations of juvenile mortality.

The results showed that population structure of *C. floribundus*, a long-lived pioneer tree species with rapid growth, varied between areas with contrasting levels of human disturbance but not with the level of natural disturbance. To our knowledge, the present study is the first to assess the differences between natural and human-modified forests in the distribution patterns of a pioneer tree species that shows no preference regarding gap size or gap age under natural forest conditions. It appears that the main ecological processes that determine the pioneer species distribution in natural and human-modified forests are similar, that is, responses to light associated with biotic interactions, seed dispersal (limited), and colonization. However, different factors or conditions generated by these contrasting levels and types of disturbances seem to change the way the ecological processes act on the species present. Hence, while the relative shade tolerance of *C. floribundus* allows this species to occupy a broad range of gap sizes in the primary forest, the unavailability of favorable sites in the early successional forest for juvenile growth and its ability to cope with herbivory under reduced interspecific competition may contribute to the high juvenile density found in this forest. Likewise, the colonization of a large open area created by intensive land use and with possible barriers to plant establishment associated with a limited seed dispersal decreases the density of trees in the early successional forest, whereas the distance from the source of seeds and barriers of seed dispersal decreases the density of juveniles in the primary forest.

## Appendix

### Appendix 1

Relative growth rate (RGR, cm cm^−1^ year^−1^) calculated as (ln DBH_*t+1*_* – *ln DBH_*t*_)/Δ*t* and plotted as a function of initial diameter at breast height (DBH_*t*_, cm) for trees of *Croton floribundus* Spreng. in the permanent plot at Caetetus Ecological Station (CES) censused in 2005 (*t*) (Rodrigues 2005) and 2010 (*t + 1*) (T. E. Barreto, unpubl. data) (*n* = 310).

## Appendix

### Appendix 2

Means ± SE of height (cm) for juveniles of *Croton floribundus* Spreng. sampled in canopy gaps in the permanent plot at Caetetus Ecological Station (*n* = 70) and in the early successional forest at Torrão de Ouro Farm (*n* = 70). Different letters indicate means statistically different by two-sample *t-* and Mann–Whitney tests with 1% and 5% of significance, respectively.

**Table:**

	Primary forest	Early successional forest
Height (cm)	21.8 ± 0.9a	17.8 ± 0.7b
